# Endoscopic ultrasound-guided NOTES for fishbone removal near the pancreas

**DOI:** 10.1055/a-2589-1350

**Published:** 2025-04-29

**Authors:** Bo Li, Fangfang Guo, Hong-Tan Chen

**Affiliations:** 1 Department of Gastroenterology, the First Affiliated Hospital, Zhejiang University School of Medicine, Hangzhou, China

Natural orifice transluminal endoscopic surgery (NOTES) is a minimally invasive technique that accesses internal organs through natural orifices, eliminating external incisions. With advancements in endoscopic technology, NOTES is increasingly applied in clinical practice 1 . Here, we report a case of endoscopic ultrasound (EUS)-guided NOTES for removing a fishbone near the pancreas.

A 59-year-old man who presented with a 10-day history of epigastric pain was admitted. Physical examination showed mild tenderness in the right upper abdomen. Laboratory tests revealed elevated white blood cell count and C-reactive protein levels. He had accidentally swallowed a fishbone 2 months prior but was asymptomatic at that time.

A computed tomography scan identified pancreatic exudation and a high-density strip near the pancreatic head, indicating the presence of a foreign body. However, traditional gastroscopy and EUS with a mini-probe failed to detect it ( Fig. 1 ).

**Fig. 1:**
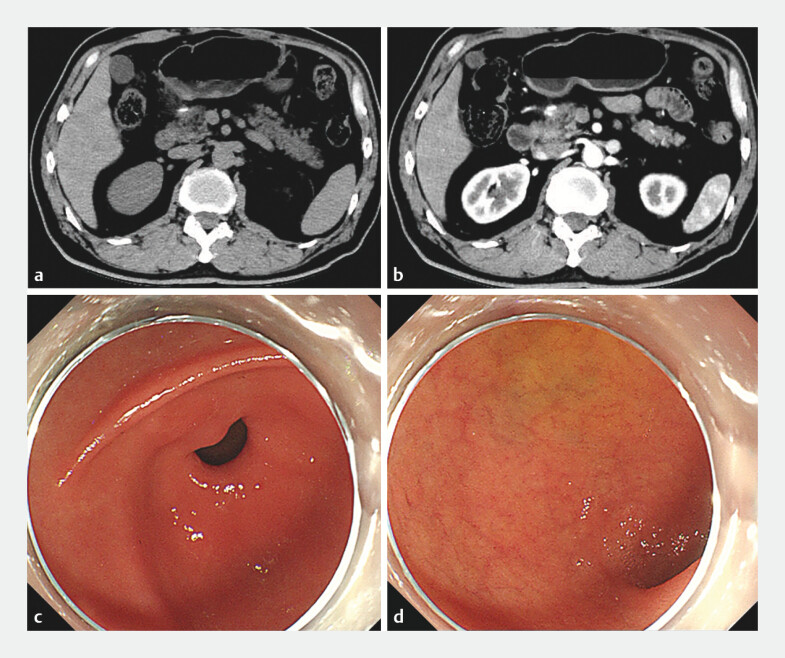
Abdominal computed tomography (CT) and gastroscopy images before the endoscopic procedure. **a, b** Abdominal plain ( **a** ) and contrast-enhanced ( **b** ) CT scans suggested a high-density foreign object in front of the pancreatic head. **c, d** Gastroscopy did not reveal any foreign body or ulcer in the antrum ( **c** ) or duodenum ( **d** ).

A linear-array echoendoscope (GF-UCT260; Olympus, Tokyo, Japan) was employed, revealing a hyperechoic foreign body adjacent to the pancreatic head. Given the foreign body’s penetration through the gastric wall and the patient’s pain, intervention was necessary. Conventional surgery posed significant trauma and difficulty in locating the object, so we opted for endoscopic full-thickness resection under EUS guidance ( Video 1 ).

Endoscopic ultrasound-guided natural orifice transluminal endoscopic surgery for fishbone removal near the pancreas.Video 1

The gastric wall was incised to the serosal layer with a HybridKnife (I-Type I-Jet; Erbe Elektromedizin GmbH, Tübingen, Germany). After relocating under EUS guidance and carefully separating the surrounding tissue, the fishbone (3 cm) was successfully removed ( Fig. 2 ). Post-procedure, the patient received anti-inflammatory treatment and recovered uneventfully. He was discharged 5 days later and showed no complications at follow-up ( Fig. 3 ).

**Fig. 2:**
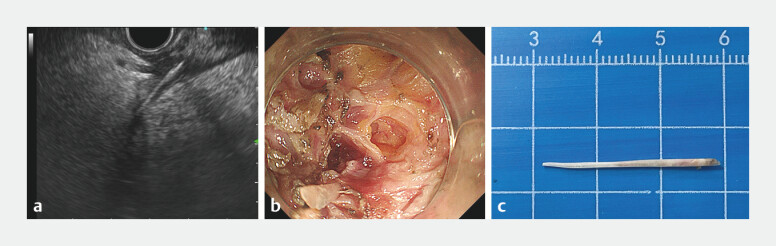
Endoscopic ultrasound (EUS)-guided endoscopic full-thickness resection to remove the foreign body. **a** Linear-array EUS scan revealed a hyperechoic, strip-shaped foreign body near the pancreatic head. **b** Following careful incision under ultrasound guidance, the foreign body was successfully exposed. **c** A fishbone approximately 3 cm long was removed successfully.

**Fig. 3:**
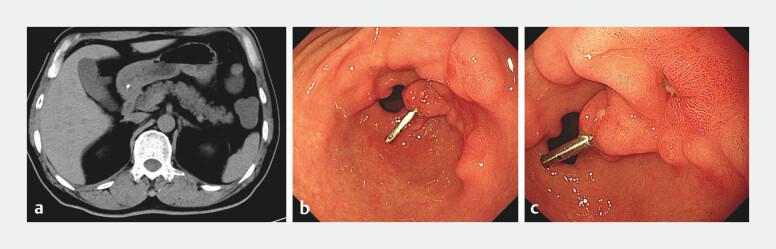
Computed tomography (CT) and gastroscopy images at the 3-month follow-up. **a** CT showed no obvious abnormalities. **b, c** Gastroscopy showed that the surgical region had mostly healed, with a residual superficial ulcer and a titanium clip.

Accidental ingestion of foreign bodies is common, but migration into the abdominal cavity, particularly near the pancreas, is rare and serious 2 . This case indicates the utility of EUS-guided NOTES for safely removing intra-abdominal foreign bodies, reducing the need for conventional surgery, and minimizing postoperative complications.

Endoscopy_UCTN_Code_TTT_1AO_2AL

## Notes

Endoscopy E-Videos https://eref.thieme.de/e-videos
*E-Videos* is an open access online section of the journal *Endoscopy* , reporting on interesting cases and new techniques in gastroenterological endoscopy. All papers include a high-quality video and are published with a Creative Commons CC-BY license. Endoscopy E-Videos qualify for HINARI discounts and waivers and eligibility is automatically checked during the submission process. We grant 100% waivers to articles whose corresponding authors are based in Group A countries and 50% waivers to those who are based in Group B countries as classified by Research4Life (see: https://www.research4life.org/access/eligibility/ ). This section has its own submission website at https://mc.manuscriptcentral.com/e-videos .
